# Effects of virtual reality-based feedback on neurofeedback training performance—A sham-controlled study

**DOI:** 10.3389/fnhum.2022.952261

**Published:** 2022-08-12

**Authors:** Lisa M. Berger, Guilherme Wood, Silvia E. Kober

**Affiliations:** ^1^Institute of Psychology, University of Graz, Graz, Austria; ^2^BioTechMed-Graz, Graz, Austria

**Keywords:** EEG, virtual reality, neurofeedback, power, SMR

## Abstract

Electroencephalography-neurofeedback (EEG-NF) has become a valuable tool in the field of psychology, e.g., to improve cognitive function. Nevertheless, a large percentage of NF users seem to be unable to control their own brain activation. Therefore, the aim of this study was to examine whether a different kind of visual feedback could positively influence NF performance after one training session. Virtual reality (VR) seems to have beneficial training effects and has already been reported to increase motivational training aspects. In the present study, we tested 61 young healthy adults (mean age: 23.48 years; 28 female) to investigate, whether 3D VR-based NF training has a more beneficial effect on the sensorimotor rhythm (SMR, 12–15 Hz) power increase than a mere 2D conventional NF paradigm. In the 3D group, participants had to roll a ball along a predefined path in an immersive virtual environment, whereas the 2D group had to increase the height of a bar. Both paradigms were presented using VR goggles. Participants completed one baseline and six feedback runs with 3 min each, in which they should try to increase SMR power over Cz. Half of the participants received real feedback whereas the other half received sham feedback. Participants receiving 3D VR-based feedback showed a linear increase in SMR power over the feedback runs within one training session. This was the case for the real as well as for the sham 3D feedback group and might be related to more general VR-related effects. The 2D group receiving the conventional bar feedback showed no changes in SMR power over the feedback runs. The present study underlines that the visual feedback modality has differential effects on the NF training performance and that 3D VR-based feedback has advantages over conventional 2D feedback.

## Introduction

Virtual reality (VR) developed to be not only a tool for the gaming industry, but also gained significant medical and psychological relevance. Highly immersive 3D virtual environments have already been used in therapeutical sessions so as to attenuate phobic manifestations (Horváthová and Siládi, 2016) and in stroke patients to support neurorehabilitation in combination with neurofeedback (NF) trainings (Vourvopoulos et al., 2019). In NF, users should learn how to alter their own brain activity. Most frequently, electroencephalography (EEG) is used to record brain activity, which is then pre-processed in real-time and fed back to the user via visual, auditory, or vibro-tactile cues.

In the present study, we focused on sensorimotor rhythm (SMR)-based feedback because of the long tradition of SMR NF (e.g., Sterman, 1996, 2000) and because it is one of the most widely used NF protocols to improve, e.g., cognitive function (for a review see Gruzelier, 2014a). Previous research of our group indicates that increasing SMR amplitude by means of NF training leads to a reduction of sensorimotor interference, which in turn might lead to improved stimulus processing capabilities and consequently to improvements in cognitive performance (Kober et al., 2015; Reichert et al., 2016). However, although SMR NF is frequently used in both NF research and practice, there are open questions concerning, e.g., the effects of feedback design (Enriquez-Geppert et al., 2017). The question of the impact of feedback design is also relevant to other NF training protocols, such as Theta/Beta, Alpha, or slow cortical potential (SCP) training, but SMR (12–15 Hz) is particularly associated with physical relaxation and mental alertness, which could be achieved with our 3D group. Slowly moving through a light-flooded forest environment might induce a physically relaxed state while focusing on the ball in the VR environment might foster focused attention at the same time. Because of these reasons, we decided to use a SMR-based NF training protocol in the present investigation. Effects of VR feedback on other EEG frequencies is a matter of future investigation.

In most NF designs, visual feedback is used, showing bars that increase and decrease in its height, depending on the brain activation (Doppelmayr and Weber, 2011; Marzbani et al., 2016). However, similar simple and little engaging designs can make feedback sessions demotivating and tiring, wherefore it seems desirable to define more engaging feedback designs. Virtual reality seems to be a desirable tool to tackle this issue, as previous studies could already suggest (Kober et al., 2016, 2017a; Vourvopoulos et al., 2019). Chronic stroke patients with motor-impairment received NF in a VR and reported high enjoyability and the wish to continue with the training even over the fixated period (Kober et al., 2016). This shows quite well that the type of feedback can increase enjoyability and therefore training-motivation, which is in turn positively associated with NF performance (Nijboer et al., 2008; Kleih et al., 2010; Hernandez et al., 2018). Kober et al. (2016) also found higher self-reported motivation of stroke patients that received 3D feedback in the VR. Additionally, patients also reported a higher feeling of control and more interest compared to patients training with the traditional 2D paradigm.

Another important point in the construction of NF studies is the implementation of control groups. In training studies, control conditions are an important part for reliable feasibility. Control groups help investigating, whether treatment effects are due to a successful training or are just a product of time or the mere believe the training would help (Thibault et al., 2015). As NF is a training intervention, it is therefore very important to consider sham feedbacks. There are differential hints in the literature, whether or not NF entails certain placebo effects. A study on migraine, for example, divided their migraine patients into a NF and into a sham group. The sham group reported a reduction of tension and anxiety, but the reported total frequency of headaches was not influenced, which also speaks for placebo (Arina et al., 2017). Another study that found both effects reported that a training for increasing upper alpha resulted in an increase of the power only for the NF group but cognitive improvement also in the sham group (Engelbregt et al., 2016). Especially due to these different results, sham groups serve as very important control factors in NF studies.

The aim of the present study was to combine NF with VR feedback to reveal its potential positive effects on NF performance (the ability to up-regulate SMR power within one session of NF training) and expand findings of previous VR-based NF studies. As there is great need of including control groups in NF trainings, having two different groups with both real and sham feedback helps gaining more insight in NF effects (Thibault et al., 2016; Enriquez-Geppert et al., 2017; Ros et al., 2020). Also, for both groups immersive VR environments have been used instead of using a computer or projection screen for the 2D paradigm, which has been only rarely performed previously (Berger and Davelaar, 2018).

Hence, we created comparably immersive designs for both groups to compare a traditional visual feedback scenario (vertically moving bars) with a 3D VR scenario (moving ball in forest environment), expecting the 3D VR scenario to result in better NF performance, compared to the 2D design. Additionally, to control for unspecific or placebo effects, we compared real feedback with sham feedback, expecting the real feedback group to have a higher NF performance. We also calculated exploratory EEG coherence analyses, which can be found in the Supplementary material B.

## Materials and methods

### Participants

Seventy-six healthy participants were tested in this study and were randomly assigned to one of four groups, 2D real or sham and 3D real or sham (Table 1). Inclusion criteria were the absence of neurological or psychiatric diseases and age of participants to be between 18 and 34 years old. Eight participants were excluded due to bad EEG data quality, technical problems and not fulfilling inclusion criteria. Further seven participants had to be excluded during analyses due to outliers in power-values (≥ 3 SD), three are SMR, three are Beta and one is Theta. Hence, 61 subjects (28 female; mean age = 23.48 years, SD = 3.49) remained for further analysis. All participants gave their written informed consent. The study was approved by the local ethics committee of the University of Graz, Austria and is in accordance with The Code of Ethics of the World Medical Association (Declaration of Helsinki) for experiments involving humans (World Medical Association, 2013).

**TABLE 1:** Description of the cohort distribution in all four groups.

Group	Real	Sham
3D	13 (7 female)	15 (7 female)
2D	17 (6 female)	16 (8 female)

### Neurofeedback training

Electroencephalography data was recorded with the gUSBamp RESEARCH EEG-amplifier from g.tec medical engineering with a sampling rate of 256 Hz. Signal was measured via 16 sintered Ag/AgCl passive ring electrodes. A conductive, liquid gel was used for an ideal impedance and signal quality. All electrodes where directly referenced against left mastoid. The ground electrode was placed at FPz, a further reference channel was placed right mastoid to be able to calculate a linked mastoid reference during offline data analysis. Impedances of references and head electrodes were held below 5 kΩ and electrooculogram (EOG) electrodes below 10 kΩ. Used electrodes placed according to the 10–20 system (Jasper, 1957) were F3, Fz, F4, C3, Cz, C4, CPz, P3, Pz, P4, O1, and O2, as well as three EOG electrodes, reference electrodes placed left and right mastoid and a ground electrode. Cz was used to give feedback as SMR primarily shows over the sensorimotor areas.

The NF sessions consisted of a baseline and six feedback runs of 3 min each where the participants received visual feedback. During the baseline run, participants should watch the paradigm moving by itself and relax and were instructed to not try to influence it. Then the individual means for SMR (12–15 Hz) for each person were calculated during online data processing based on the data recorded during the baseline run as a threshold for the NF training. Also, Theta (4–7 Hz) and Beta (16–30 Hz) were included for artifact control, as Theta is associated with blinking and eye movements, whereas Beta is associated with muscle artifacts (Tatum et al., 2011; Kober et al., 2017a,^2020^). So, the mean plus one standard-deviation were calculated for Theta and Beta thresholds additionally. Participants were instructed to be physically relaxed, blink as seldom as possible to keep both Theta and Beta as low as possible. Also, participants were instructed to increase SMR by being mentally focused and physically relaxed, as this is the state where SMR shows.

For the visualization of the NF, the HTC Vive Pro-System was used. The paradigms were programmed using the game engine Unity 3D, Version 2018.3.1.4 and for the visualization in the VR system, SteamVR has been used, wherefore the lab streaming layer LSL4Unity plugin, freely available at https://github.com/labstreaminglayer/LSL4Unity, was implemented to stream the incoming EEG data from OpenViBE 2.2.0. This is also an open-source software for neuroscientific research, which is mostly used for real-time online data preprocessing as in NF and as an interface between incoming signal and feeding it into the paradigm after preprocessing. Both the 3D and 2D paradigms are described in the following.

The sham group had the same experimental set-up and got the same instruction. However, they did not get feedback about their own brain activation, but the activation from another person collected in a previous EEG SMR NF study. Participants did not know that there were several different groups and the experimenter did not know whether the participants received real or sham feedback and had fixed instructions.

Before and after the intervention participants had to fill out subjective questionnaires on motivation, subjective feeling of presence and flow, on usage of technology and cybersickness. The results of which are presented elsewhere (Berger et al., 2021). The present paper focusses on effects on NF performance and concomitant changes in other EEG parameters.

We also included the CRED-nf checklist, which was developed to improve the reporting and experimental design standards of NF studies, in the Supplementary material A, referring to where in our paper which relevant information on the design can be found (Ros et al., 2020).

### 3D condition

**FIGURE 1:**
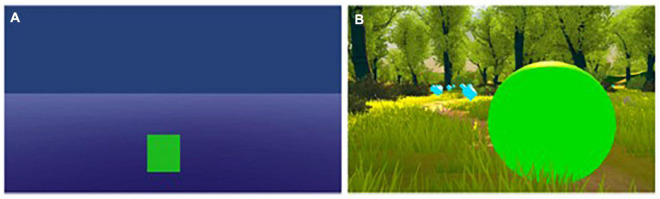
**(A)** 2D virtual reality (VR) paradigm. **(B)** 3D VR paradigm. Both were presented via the HTC-Vive Pro head-mounted VR goggles.

The 3D paradigm consisted of a very light-flooded forest environment, which was a free Demo retrieved from the Unity Asset Store (Fantasy Forest Environment from TriForge Assets). The users saw the environment in first-person perspective and saw in front of them a green ball, that would roll along a predefined path and collect light blue floating cubes, which marked the path. When the ball was moving, the camera was moving behind the ball and would rotate with the ball when taking curves, so that the user would have the sensation of following the ball on its way. The ball would move in a constant speed every time the EEG power exceeded the predefined SMR-threshold, which was set for every participant individually based on an initial baseline run. The ball would stand still when the signal was below this threshold. Additionally, the ball would change its color to red and stand still, whenever Beta or Theta were over the individually set thresholds and therefore the artifacts were too high, for example due to eye movements/blinking or muscle artifacts. This means the ball would only move when both the artifact and SMR values would be in the desired, predefined range (see Figure 1B).

### 2D condition

The participants in the 2D group saw a dark-blue background and a dark floor on which a green bar was placed that could increase and decrease in its size. The bar itself was completely flat, meaning it had no depth, therefore seemed to be 2D (see Figure 1A). The bar would increase in height every time the user exceeded the individually predefined thresholds. It had a predefined maximum and minimum height, so that the bar would not disappear out of sight of the participants, so they did not have to move their heads during the feedback runs. When the bar reached its maximum height, it would stay there until the SMR power was below the set threshold. The bar would sink every time the power-values were below the set threshold but did not completely disappear in the ground so that even if it was on its lowest, a narrow line could be seen to identify its color. This is because the bar could also change its color to red and stay still each time the artifacts exceeded their thresholds. Therefore, the bar would only increase in height, when both the SMR and artifact values would be in the predefined range (see Figure 1A).

### Offline electroencephalography analysis

For offline data processing, the Brain Vision Analyzer software was used (version 2.2, Brain Products GmbH, Munich, Germany). At the beginning, a 50 Hz notch filter was applied, as well as a high pass filter of 1 Hz to eliminate low frequencies such as large drifts and a lowpass filter of 40 Hz to eliminate high frequencies, were also applied (Brain Products GmbH, 2008). After filtering, raw data was manually inspected for larger artifacts, such as big muscle artifacts or heavy drifts. Data was then referenced to a linked mastoid reference to rule out hemisphere effects, as the left mastoid was the primary reference electrode. Then, the semi-automatic independent component analysis (ICA) ocular correction followed. Blinks as well as eye movement components were eliminated in this step. As a last preprocessing step, a second semi-automatic data inspection was made to exclude possible artifacts that survived the other preprocessing steps (Criteria for rejection: maximum allowed voltage step of 50 μV/ms, maximum allowed difference between values in a segment was 200 μV, amplitudes ± 120 μV, lowest allowed activity in 100 ms intervals was 0.5 μV, artifacts were marked 200 ms before and after emergence). Twelve channels were used here: F3, Fz, F4, C3, Cz, C4, CPz, P3, Pz, P4, O1, O2.

In the next steps, power in the range of the frequency bands SMR (12–15 Hz), Beta (16–30 Hz) and Theta (4–7 Hz) were extracted in the BrainVision Analyzer using complex demodulation (Draganova and Popivanov, 1999). Data was segmented into 1 s intervals and segments with artifacts were removed.

### Statistical analysis

To evaluate between- and within-group differences over all seven runs concerning the dependent variable SMR power increase over electrode position Cz, a linear mixed effect model with three fixed linear effects (3D vs. 2D, real vs. sham and feedback runs) was calculated (Type I Analysis of Variance with Satterthwaite’s method). The same method was also used for Beta and Theta power increase. Subjects and individual regression slopes across runs were included in the model as crossed random effects (Baayen et al., 2008). Mixed effect modeling was performed in R (Bates et al., 2015), freely available at http://cran.r-project.org. The lmer4 and lmerTest packages were used (Bates et al., 2015).

We will refer to the factor 3D vs. 2D as “group” or “feedback group” and the feedback type real vs. sham we will refer to as “condition” in the following sections for clarification.

## Results

### Effects of feedback modality (3D vs. 2D) on SMR power

**FIGURE 2:**
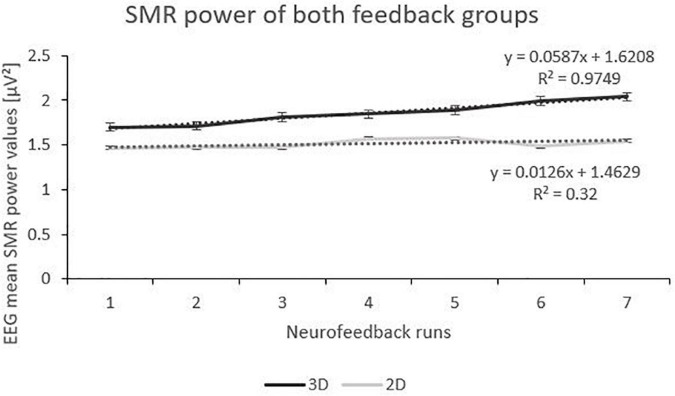
Trend of SMR Power over the Feedback Runs for 3D and 2D Groups with the linear trendlines of each group. *R*^2^ represents the respective explained variance of SMR power. Error Bars show Standard Error.

The linear mixed effect model for the dependent variable SMR power showed a significant within-subject factor “runs” and a significant interaction effect between “feedback group” and “runs” (Table 2). Post-tests (separate mixed effect model analysis per feedback group) revealed a significant main effect runs (*F*(1,166) = 29.29, *p* < 0.000, η_*p*_^2^ = 0.01; see Figure 2) for the 3D feedback-group, indicating a linear increase in SMR power over feedback runs in the 3D feedback-group, whereas the mixed effect model analysis for the 2D group revealed no significant effects (*F*(1,196) = 2.64, *p* = 0.106).

**TABLE 2:** *F* statistics of the linear mixed models for electroencephalography (EEG) power of SMR, Theta, and Beta.

Analysis	Factors	*F* (1,57)	*p*	η _*p*_^2^	sig.
SMR power	Group × runs	12.46	0.001	0.004	***
	Runs	26.91	0.000	0.006	***
	Group	2.23	0.141		
	Condition	0.81	0.373		
	Group × condition	0.35	0.558		
	Condition × runs	0.25	0.620		
	Group × condition × runs	0.02	0.894		
Theta power	Group × runs	4.02	0.046	0.000	*
	Runs	21.54	0.000	0.000	***
	Group	3.80	0.056		.
	Condition	2.46	0.122		
	Group × condition	0.27	0.608		
	Condition × runs	5.76	0.017	0.006	*
	Group × condition × runs	0.54	0.465		
Beta power	Group × runs	(1, 362) 9.23	0.003	0.001	**
	Runs	(1, 362) 0.20	0.658		
	Group	(1, 57) 0.09	0.767		
	Condition	(1, 57) 0.55	0.462		
	Group × condition	(1, 57) 0.23	0.635		
	Condition × runs	(1, 362) 1.39	0.239		
	Group × condition × runs	(1, 362) 0.24	0.621		

The factor group indicates feedback group (3D vs. 2D) and condition indicates real vs. sham feedback. “***” *p* < 0.001, “** “*p* < 0.01, “*”*p* < 0.05, “.”*p* = 0.05.

### Effects of feedback modality (3D vs. 2D) on Theta and Beta power

**FIGURE 3:**
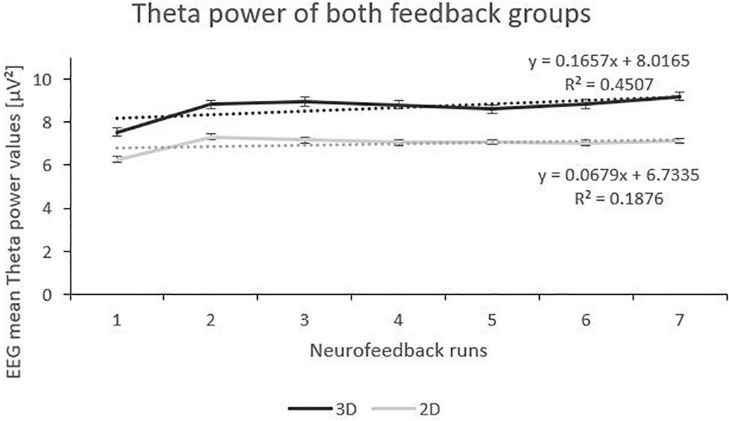
Trend of Theta Power over the Feedback Runs for 3D and 2D Groups with the linear trendlines of each group. *R*^2^ represents the respective explained variance of Theta power. Error bars show Standard Error.

Analysis of the Theta frequency band showed fixed main effects “runs”. Both 3D and 2D group showed an increase of Theta power over the feedback runs but on a between-group level, the 3D group showed a tendentially higher level of Theta (see Figure 3). There was a significant interaction effect of the fixed main effects “group” and “runs”, with a greater increase in the 3D group. Also, there was an interaction effect of the fixed main effects “condition” (real vs. sham) and “runs”, with a significant increase in the sham groups (*F*(1,184) = 23.06, *p* < 0.000, η_*p*_^2^ = 0.05) compared to the real feedback groups (*F*(1,178) = 2.45, *p* = 0.119). No other effects where significant (see Table 2).

**FIGURE 4:**
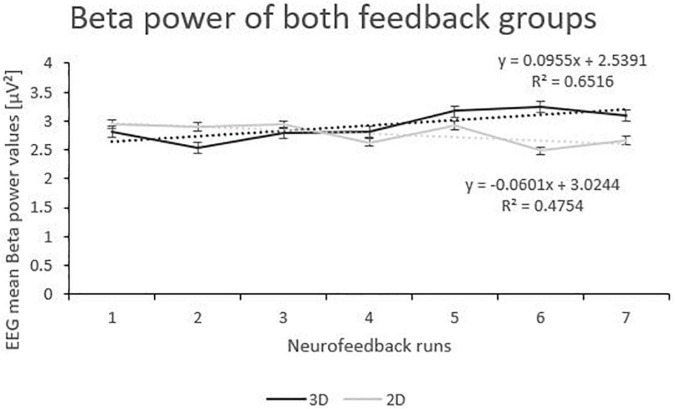
Trend of Beta Power over the Feedback Runs for 3D and 2D Groups with the linear trendlines of each group. *R*^2^ represents the respective explained variance of Beta power. Error bars show Standard Error.

Beta power showed an interaction effect of “group” and “runs”. Post-tests (separate mixed effect model analysis per feedback group) revealed that the 3D group significantly increased Beta power over the feedback runs (*F*(1,166) = 7.39, *p* < 0.001, η_*p*_^2^ = 0.001), while the 2D group did not (*F*(1,196) = 2.70, *p* = 0.102; see Figure 4). No other effects where significant (see Table 2).

## Discussion

### 3D vs. 2D feedback on electroencephalography power

In the present study, participants had to regulate the movement of either 3D or 2D target objects in a VR scenario during one NF training session with either real or sham feedback to investigate the relevance of visual feedback on NF performance. Additionally, we did exploratory analyses on EEG coherence (see Supplementary material B) as changes due to NF are not isolated to the trained brain region or frequency band, but goes hand in hand with electrophysiologic changes in surrounding brain regions and even other frequency bands (Gruzelier, 2014b; Kober et al., 2015, 2020).

The 3D group showed a linear increase in SMR power while the 2D group did not show significant changes in SMR power across feedback runs. This indicates that 3D visual feedback has a greater beneficial effect on SMR power increase over traditional 2D visual feedback. However, this might speak for a more unspecific effect of VR itself rather than a NF related specific effect, since both real and sham 3D feedback groups showed this increase in SMR power. This result is in accordance with previous studies comparing NF effects of 2D- or 3D forms of feedback. In one study on neural learning half of the participants had to increase their upper alpha in a 3D virtual environment and the other half in a 2D virtual environment (Berger and Davelaar, 2018). The NF task was to let either a 3D or a 2D object levitate, presented via a head-mounted VR-system. Neurofeedback training took place on 5 days and the aim was to measure its effects on cognitive processes (Stroop task) depending on the type of feedback. Statistical analyses showed that only the 3D group could modulate upper alpha voluntarily. Effects were not significant for the 2D group. They argue that attention, immersion and a higher learning curve might be driving components of 3D feedback (Berger and Davelaar, 2018), which are VR unspecific effects. Another study aiming at reducing stress through four sessions of frontal alpha NF training also showed that 3D visualizations resulted in more beneficial effects on frontal alpha and stress reduction than 2D visualizations. They used a gamification approach–in the 2D paradigm, the game “Bugz Shooter” was used, where participants had to shoot bugs with water bubbles depending on frontal alpha asymmetry. For the 3D group, a car driving game was presented, and the car would drive depending on the alpha asymmetry (Hafeez et al., 2019). As the studies did not include control groups one cannot differ between NF-specific effects and unspecific 3D/VR-related effects. It could also be that visually more complex designs have better effects in NF studies (Alimardani et al., 2013). SMR also seems to depend on visual attention processes (Sterman, 1996), wherefore visually more complex NF designs, such as 3D designs, could have beneficial effects on increasing SMR during NF training.

Psychological factors seem to be highly relevant in SMR power increase. A meditation study could show, that NF meditation in a VR environment helped the users reach deeper relaxation compared to the control groups (Kosunen et al., 2016). Hence it seems, that relaxation could also play a relevant role in NF success. In the present study, the 3D paradigm could have been more relaxing to participants than the 2D paradigm. The setting of the former is a forest environment, and the target object could either roll forward or stand still, that means you could not loose progress. The 2D paradigm, however, took place in a dark space and the bar could decrease in height, which might have been more stressful, as it seemed like a loss of progress. As SMR also is an indicator for physical relaxation (Marzbani et al., 2016) it might be that people from the 3D group, who showed higher SMR power increase, experienced the training as more relaxing. Although participants were instructed to keep the Theta frequency band as low as possible, an increasement was shown in both groups, but Theta had a tendentially higher level in the 3D group and increased stronger in the 3D group over the feedback runs compared to the 2D group. Theta is also associated with meditative and deep states (Marzbani et al., 2016). So, it seems, that participants in the 3D group were more relaxed than those from the 2D group. This is insofar coherent with the main results, as SMR power is also associated with physical relaxation (Marzbani et al., 2016). This shows that it is important to assess user experience in NF studies. As reported in Berger et al. (2021), we also measured some further psychological factors such as subjective feeling of presence and flow. However, no group differences in those domains were found. This might be due to the study design itself. Since the movement of the ball in the 3D condition or the movement of the feedback bar in the 2D condition stopped whenever the participants produced artifacts (which led to an increase in theta and beta control frequencies), the emergence of flow experience might be disturbed. Additionally, both paradigms were presented using the VR goggles and were equally immersive, which might automatically increase the feeling of presence (Ijsselsteijn et al., 2001; Kober et al., 2012). This shows that for future studies it is important to investigate the subjective experience more thoroughly by including measures for enjoyment and relaxation to differentiate better between the groups.

We also found an unspecific increase for the sham feedback condition, compared to the real feedback condition, independently from the feedback group (3D or 2D). As both conditions did not differ in any other frequency band or condition, this result speaks for an unspecific effect. It can be, that hence 3D paradigms are beneficial for SMR trainings, inducing relaxation over light-flooded forest environments and fostering of attention through the focus on the ball. Effects of VR feedback on other EEG frequencies is a matter of future investigation.

Furthermore, even though participants were instructed to reduce the beta frequency band, we observed only for the 3D group and not for the 2D group a significant increase over the feedback runs for Beta. As already mentioned, concomitant changes of other frequency bands, such as Beta, have already been found previously (Ghoshuni et al., 2012; Kober et al., 2015), but are not reported in most other studies (Ros et al., 2020). Hence, band specificity is still an open topic in NF literature and needs to be investigated further.

As NF studies using VR are still relatively new, very different forms of visualizations of 2D and 3D paradigms can be found in the existing literature. Some research groups present the same scenario in both groups, only with a different render, so the same picture seems 3D for one group and for the other group only 2D (Li et al., 2020). Similar to this kind of approach, some studies present the same environment, just with different target objects, where one is 3D and the other is not (Berger and Davelaar, 2018). Therefore, unintentional moderating variables such as different brightness or color-intensities that might influence attentional effects can be ruled out more easily. This factor can pose a limitation in the present study. The 3D paradigm was visibly brighter than the 2D paradigm, which could result in a greater wakefulness and by contrast more tiredness in the 2D group over the course of the NF session. This effect, however, can be ruled out as Theta, which also can be an indicator for sleepiness (Marzbani et al., 2016), was not higher in the 2D group and did not increase more than in the 3D group.

Consequently, it can be reasoned that the 3D paradigm resulted in a better SMR NF performance, than the 2D group

### Sham feedback vs. real feedback

Interestingly, the aforementioned effects were independent from getting real or sham feedback. It did not matter, whether the participants got their own brain activation visualized or that from another person.

Double-blind sham control groups are still relatively sparse in the literature (Ramos-Murguialday et al., 2013; Park et al., 2014; Schabus et al., 2014; Kober et al., 2015), but discussions on possible placebo effects have been quite extensive and revealed some interesting explanations on why such effects might emerge. Thibault et al. (2017) suggested that very specific NF-behavior, such as SMR power increase, can be driven by unspecific factors. Witte et al. (2018) added that such unspecific results are often unjustly labeled as placebo effects, where they rather are the result of for example psychological reactions on the experiment. Motivation has for example already been reported as a driving factor of SMR power increase (Nijboer et al., 2008; Kleih et al., 2010; Hernandez et al., 2018). Some other psychological factors that might contribute to SMR power increase that have already been reported in previous studies were among others subjective control beliefs (Witte et al., 2013), mood (Nijboer et al., 2008), or immersion (Magosso et al., 2019). These are all factors that can as well be present in sham feedback groups. Participants can be motivated for the training sessions and feel confident to succeed in increasing the relevant frequency band powers regardless of the feedback visualization method (Witte et al., 2018). Expectations of the participants implied by specific instructions or mock interventions could also affect NF training performance (e.g., Kober et al., 2018). In the present study, all four groups got the exact same instructions and standardized answers to questions, so instructional effects could be ruled out. Further it is pointed out, that placebo might be one kind of learning, especially because the tasks and aims are mostly equal to those receiving real feedback. Which again strengthens the idea, that NF is a complex set of psychophysiological factors (Kober et al., 2018; Ros et al., 2020).

Another factor, that seems to play a role in SMR power increase, is the number of training sessions. Many NF studies showing stronger beneficial effects in real feedback groups compared to sham groups performed about 10 or more feedback sessions. A review on attention deficit hyperactivity disorder (ADHD) suggested that NF may not have more beneficial effects than sham feedback, due in part to methodological problems (Vollebregt et al., 2014). They propose, for example, that the effects of NF need more time in the form of more training sessions to develop. It might be that over the course of 20–30 min (average duration of one NF training session), attention and concentration will naturally increase as you become familiar with the task. Therefore, regardless of the type of feedback, an increase in SMR power would be natural and more sessions would be needed to reveal efficiency differences between sham and real feedback groups. It seems that the unspecific effects of VR have beneficial effects on NF performance and improves the training process within the first training session.

Previous studies with multiple VR-based NF training sessions reporting significant differences did not include any control groups (Kober et al., 2017b; Berger and Davelaar, 2018; Hafeez et al., 2019). Hence, it is not sure whether the differences are due to non-specific effects of the VR paradigms rather than the NF training itself. For further VR NF studies it would therefore be important to include both, several training sessions and control groups. Since SMR NF training has positive effects on cognitive function (Zoefel et al., 2011; Gruzelier, 2014a) it would be interesting to investigate in further studies, whether such unspecific influences of VR on the NF performance would be sufficient to positively affect cognition as well. It has already been shown, that unspecific effects can reduce tension and anxiety in migraine patients (Schabus et al., 2017) and result in cognitive improvements also in sham groups (Engelbregt et al., 2016).

## Conclusion

Previous results (Gruzelier et al., 2010; Kleih et al., 2010; Hammer et al., 2012; Kober et al., 2017a) as well as the present results show that NF success can be driven by several different unspecific psychological and technical effects, wherefore also sham feedback can result in positive feedback effects. However, this does not necessarily support the argument that NF would be just a placebo but rather that feedback protocols should be reported and designed more carefully to find out possible underlying specific and unspecific effects. 3D VR NF training has a positive impact on SMR NF training performance compared to a mere 2D design (Ros et al., 2020). These results show the importance to adapt NF training paradigms to make training more appealing and therefore more efficient and investigate those results further in future studies.

## Data availability statement

Ethical restrictions prohibit the authors from making the dataset publicly available. Data are available from the corresponding author (LMB) after contacting the Ethics Committee of the University of Graz (ethikkommission@uni-graz.at) for researchers who meet the criteria for access to confidential data. When the Ethics Committee of the University of Graz agrees, the readers can contact the corresponding author (LMB) (lisa.berger@uni-graz.at) to request the data. With the approval of the Ethics Committee of the University of Graz, we confirm that data will be available upon request to all interested researchers.

## Ethics statement

The studies involving human participants were reviewed and approved by the Ethics Committee of the University of Graz. The patients/participants provided their written informed consent to participate in this study.

## Author contributions

All authors were involved in designing the research and conceptualization. LMB performed the research. SEK supervised the conduction of the study. SEK and GW contributed resources, software, and/or analytic tools. SEK and LMB performed data analysis and interpretation. LMB wrote the original draft. All authors reviewed, edited, and approved the manuscript.
