# Supplementary material for: Effects of virtual reality-based feedback on neurofeedback training performance—A sham-controlled study
Source: Front Hum Neurosci. 2022 Aug 12;16:952261. doi: 10.3389/fnhum.2022.952261 (PMC9411512; doi:10.3389/fnhum.2022.952261)
Supplement: Supplementary file 1 [file Data_Sheet_1.docx]

**Supplementary Material**

Title: Effects of VR-based feedback on neurofeedback training performance
– a sham-controlled study

*Lisa M. Berger^1*^, Guilherme Wood^1,2^, Silvia E. Kober^1,2^

^1^ Institute of Psychology, University of Graz, Universitaetsplatz 2/III, 8010 Graz, Austria

^2^ BioTechMed-Graz, Mozartgasse 12/II, 8010 Graz, Austria

**Supplementary material A**

**Table A**

*Consensus on the reporting and experimental design of clinical and cognitive-behavioural neurofeedback studies (CRED-nf checklist) best practices checklist (Ros et al., 2020)*

| **Domain** | **Item #** | **Checklist item** | **Reported in** | **Comments** |
| --- | --- | --- | --- | --- |
| **Pre-experiment** | | | |  |
|  | 1a | Pre-register experimental protocol and planned analyses | X |  |
|  | 1b | Justify sample size | X | Sample size was determined based on previous studies (Ninaus et al., 2015; e.g., Kober et al., 2017). |
| **Control groups** | | | |  |
|  | 2a | Employ control group(s) or control condition(s) | Methods section | Sham control group |
|  | 2b | When leveraging experimental designs where a double-blind is possible, use a double-blind | Methods section | Participants did not know there would be different groups and conditions, experimenters did not know which participant received real feedback and who received sham feedback during NF training. |
|  | 2c | Blind those who rate the outcomes, and when possible, the statisticians involved | X |  |
|  | 2d | Examine to what extent participants and experimenters remain blinded | Methods section | Participants and experimenters were blinded during NF training concerning real/sham feedback conditions. |
|  | 2e | In clinical efficacy studies, employ a standard-of-care intervention group as a benchmark for improvement | X | This was no clinical efficacy study. |
| **Control measures** | | | |  |
|  | 3a | Collect data on psychosocial factors | X | Reported in another study on VR user experience (Berger et al., 2021) |
|  | 3b | Report whether participants were provided with a strategy | Methods section |  |
|  | 3c | Report the strategies participants used | X |  |
|  | 3d | Report methods used for online-data processing and artifact correction | Methods section |  |
|  | 3e | Report condition and group effects for artifacts | Results section |  |
| **Feedback specifications** | | | |  |
|  | 4a | Report how the online-feature extraction was defined | Methods section |  |
|  | 4b | Report and justify the reinforcement schedule | Methods section |  |
|  | 4c | Report the feedback modality and content | Methods section |  |
|  | 4d | Collect and report all brain activity variable(s) and/or contrasts used for feedback, as displayed to experimental participants | Methods section & Results section |  |
|  | 4e | Report the hardware and software used | Methods section |  |
| **Outcome measures** | | | |  |
| Brain | 5a | Report neurofeedback regulation success based on the feedback signal | Results section |  |
|  | 5b | Plot within-session and between-session regulation blocks of feedback variable(s), as well as pre-to-post resting baselines or contrasts | Results section | Only one NF training session was performed |
|  | 5c | Statistically compare the experimental condition/group to the control condition(s)/group(s) (not only each group to baseline measures) | Results section |  |
| Behaviour | 6a | Include measures of clinical or behavioural significance, defined a priori, and describe whether they were reached | X | No clinical or behavioural measures apart from EEG and NF performance were investigated |
|  | 6b | Run correlational analyses between regulation success and behavioural outcomes | X |  |
| **Data storage** | | |  |  |
|  | 7a | Upload all materials, analysis scripts, code, and raw data used for analyses, as well as final values, to an open access data repository, when feasible | See data availability |  |

**Supplementary material B**

**EEG Coherence**

In an additional exploratory post-hoc approach, we conducted coherence analyses as it has been shown previously, that brain changes due to NF are not isolated to the trained brain region or frequency band, but goes hand in hand with electrophysiologic changes in surrounding brain regions and even other frequency bands (Gruzelier, 2014; Kober et al., 2015; Kober et al., 2020). Cognitive processes often entail the activation of bigger networks instead of only one dedicated brain activation (Ninaus et al., 2013; Emmert et al., 2016). There are few hints in the literature, that participants showing lower measures of functional connectivity of spatially divided brain areas, that is EEG coherence, have been more successful in SMR neurofeedback training sessions (Kober et al., 2020).

The SMR is generated in somatosensory relay nuclei of the thalamus and emerges in a motionless yet attentive state of being. In this state, the motoric output and somatosensory information flow in the thalamocortical loop are suppressed. Hence, higher SMR values would reduce information flow to its surrounding brain areas, wherefore we would expect a concomitant decrease in SMR coherence measures during successful up-regulation of SMR power during neurofeedback training (Kober et al., 2015).

For the coherence analysis we calculated the imaginary part of coherence for the channel pairs. The imaginary coherence differs between true connectivity, where signals with time-lag are from spatially divided sources and volume conduction, assuming signals without time-lag. Therefore, proceeding from the artifact-free 1 second intervals, FFT transformation was performed epoch-wise (Hanning, window, 10%) (Nolte et al., 2004). Afterwards, the imaginary part of coherence and average coherence values were calculated for Cz-Fz, Cz-CPz and Cz-Pz in the frequency range of 12-15 Hz for each run (Kober et al., 2015; Kober et al., 2020).

For SMR coherence, we Fisher’s z transformed the values and calculated the same linear mixed effect model as for SMR power described in the main manuscript. Coherence was calculated for the adjacent channel pairs Cz-Fz, Cz-CPz and Cz-Pz. Statistical analyses showed an increase of coherence over all feedback runs for both groups for the channel pairs Cz-Pz and Cz-CPz (see Figures B1 and 2). For Cz-Fz, there was an interaction effect of group and condition (see Figure B3) with a decrease of coherence for the real 2D group (*F*(1,34)=6.47, *p* < 0.05, *η*_p_^2^ = 0.02), compared to the sham 2D group (*F*(1,24) = 0.50, *p* = .487, see Table B1).

**Figure B1**

*Trend of SMR Coherence for the channel pair Cz-Pz for the 3D and 2D groups, showing trendlines of each group. R² represents the respective explained variance of SMR Coherence. Error Bars show Standard Error.*


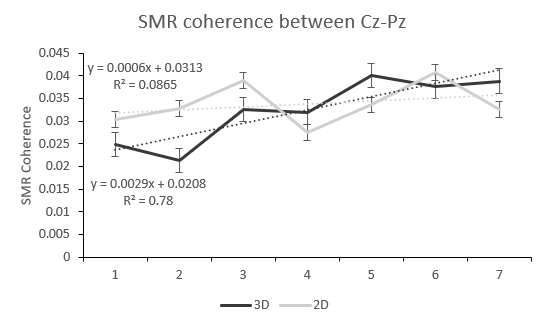


**Figure B1**

*Trend of SMR Coherence for the channel pair Cz-CPz for the 3D and 2D groups, showing trendlines of each group. R² represents the respective explained variance of SMR Coherence. Error Bars show Standard Error.*


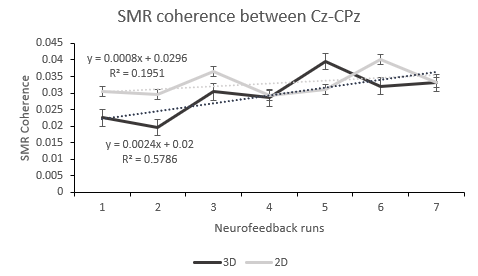


**Figure B1**

*Trend of SMR Coherence for the channel pair Cz-Fz for the 3D and 2D groups, showing trendlines of each group. R² represents the respective explained variance of SMR Coherence. Error Bars show Standard Error.*


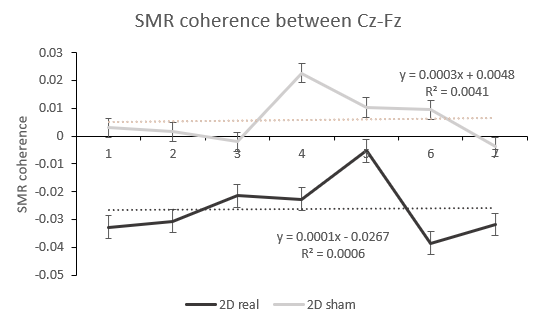


The interaction effect for the group and condition, showing a decrease of coherence for the channel pair Cz-Fz only for the real feedback group, but not for the sham feedback group is an unspecific training result. A decrease in coherence is normally associated with a better neurofeedback performance (Kober et al., 2015; Kober et al., 2020) and the real and sham feedback group in our study did not differ in their neurofeedback training performance. Also, the 2D group was not able to increase their SMR over the neurofeedback runs. The same accounts for the increase in coherence for the channel pairs Cz-Pz and Cz-CPz independently from the feedback groups.

As already mentioned in the main article, concomitant changes of other frequency bands and other brain areas are not reported in most other studies (Ros et al., 2020). Hence, activations in surrounding brain areas as a result to neurofeedback training is still an open topic in NF literature and need to be investigated further.

## Post-hoc analysis SMR Coherence

**Table B1**

F statistics for the linear mixed model of the EEG coherence values for the channel pairs Cz-Pz, Cz-CPz and Cz-Fz.

| Coherence | Factors | *F* (df) | *p* | *η*_p_^2^ | sig. |
| --- | --- | --- | --- | --- | --- |
| Cz-Pz | Group | 0.03 (1, 58) | 0.868 |  |  |
|  | Condition | 1.53 (1, 58) | 0.220 |  |  |
|  | Runs | 6.11 (1, 368) | 0.014 | 0.000 | * |
|  | Group x Condition | 2.52 (1, 58) | 0.118 |  |  |
|  | Group x runs | 3.11 (1, 368) | 0.079 |  |  |
|  | Condition x runs | 0.71 (1, 368) | 0.402 |  |  |
|  | Group x condition x runs | 0.43 (1, 368) | 0.512 |  |  |
| Cz-CPz | Group | 0.23 (1, 58) | 0.637 |  |  |
|  | Condition | 2.17 (1, 58) | 0.146 |  |  |
|  | Runs | 5.00 (1, 368) | 0.026 | 0.000 | * |
|  | Group x Condition | 1.42 (1, 58) | 0.238 |  |  |
|  | Group x runs | 1.34 (1, 368) | 0.248 |  |  |
|  | Condition x runs | 0.12 (1, 368) | 0.725 |  |  |
|  | Group x condition x runs | 0.17 (1, 368) | 0.682 |  |  |
| Cz-Fz | Group | 0.56 (1, 58) | 0.459 |  |  |
|  | Condition | 2.14 (1, 58) | 0.149 |  |  |
|  | Runs | 0.30 81, 368) | 0.582 |  |  |
|  | Group x Condition | 4.70 (1, 58) | 0.034 | 0.030 | * |
|  | Group x runs | 0.80 (1, 368) | 0.371 |  |  |
|  | Condition x runs | 0.14 (1, 368) | 0.710 |  |  |
|  | Group x condition x runs | 0.12 (1, 368) | 0.724 |  |  |

**Supplementary material C**

**Data quality in a combined VR and EEG setting**

Combining EEG set-ups with head-mounted VR-systems raises the question whether head straps could influence signal quality.

To evaluate this matter, we conducted a pilot study (Kober et al., 2021) to investigate the effects of a HMD (VR goggles) on EEG signal quality by conducting resting-state measurements with closed eyes with and without HMD.

40 healthy adults were tested during this study. Two resting measurements with closed-eyes of one minute each were performed. In one resting measurements, participants wore the HMD (VR goggles), while in the other resting measurement they did not wear the HMD (VR goggles).

The SMR power of the following electrode positions were investigated: FC3, FC4, C3, C1, C2, C4, CP3, CP4.

**Figure C1**

*SMR power for eight different electrode positions during closed-eye resting condition with and without Head Mounted Display (HMD) to indicate possible differences in EEG data quality.*


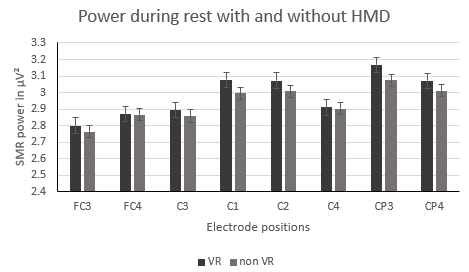


Differences were not significant (p > .44).

REFERENCES

Emmert, K., Kopel, R., Sulzer, J., Brühl, A. B., Berman, B. D., Linden, D. E. J., et al. (2016). Meta-analysis of real-time fMRI neurofeedback studies using individual participant data: How is brain regulation mediated? *Neuroimage* 124, 806–812. doi: 10.1016/j.neuroimage.2015.09.042

Gruzelier, J. H. (2014). EEG-neurofeedback for optimising performance. III: a review of methodological and theoretical considerations. *Neurosci Biobehav Rev* 44, 159–182. doi: 10.1016/j.neubiorev.2014.03.015

Kober, S. E., Neuper, C., and Wood, G. (2020). Differential Effects of Up- and Down-Regulation of SMR Coherence on EEG Activity and Memory Performance: A Neurofeedback Training Study. *Front Hum Neurosci* 14, 606684. doi: 10.3389/fnhum.2020.606684

Kober, S.E., Settgast, V., Brunnhofer, M., Augsdörfer, U., & Wood., W. (2021). Move your virtual body: differences and similarities in brain activation patterns during hand movements in real world and virtual reality. *Virtual Reality* 26, 501–511. doi: 10.1007/s10055-021-00588-1

Kober, S. E., Witte, M., Ninaus, M., Koschutnig, K., Wiesen, D., Zaiser, G., et al. (2017). Ability to Gain Control Over One’s Own Brain Activity and its Relation to Spiritual Practice: A Multimodal Imaging Study. *Front. Hum. Neurosci* 11, 1–12. doi: 10.3389/fnhum.2017.00271

Kober, S. E., Witte, M., Stangl, M., Väljamäe, A., Neuper, C., and Wood, G. (2015). Shutting down sensorimotor interference unblocks the networks for stimulus processing: an SMR neurofeedback training study. *Clin Neurophysiol* 126, 82–95. doi: 10.1016/j.clinph.2014.03.031

Ninaus, M., Kober, S., Witte, M., Koschutnig, K., Neuper, C., and Wood, G. (2015). Brain volumetry and self-regulation of brain activity relevant for neurofeedback. *Biological Psychology* 110, 126–133. doi: 10.1016/j.biopsycho.2015.07.009

Ninaus, M., Kober, S. E., Witte, M., Koschutnig, K., Stangl, M., Neuper, C., et al. (2013). Neural substrates of cognitive control under the belief of getting neurofeedback training. *Front Hum Neurosci* 7, 914. doi: 10.3389/fnhum.2013.00914

Nolte, G., Bai, O., Wheaton, L., Mari, Z., Vorbach, S., and Hallett, M. (2004). Identifying true brain interaction from EEG data using the imaginary part of coherency. *Clin Neurophysiol* 115, 2292–2307. doi: 10.1016/j.clinph.2004.04.029

Ros, T., Enriquez-Geppert, S., Zotev, V., Young, K. D., Wood, G., Whitfield-Gabrieli, S., et al. (2020). Consensus on the reporting and experimental design of clinical and cognitive-behavioural neurofeedback studies (CRED-nf checklist). *Brain* 143, 1674–1685. doi: 10.1093/brain/awaa009
